# Supplementary material for: Predicting Tolerance to Cow’s Milk Allergy in Children Using IgE and IgG4 Peptide Binding Profiles
Source: Cells. 2025 Feb 27;14(5):344. doi: 10.3390/cells14050344 (PMC11899117; doi:10.3390/cells14050344)
Supplement: Supplementary file 1 [file cells-14-00344-s001.zip › cells-3475151-supplementary.pdf]

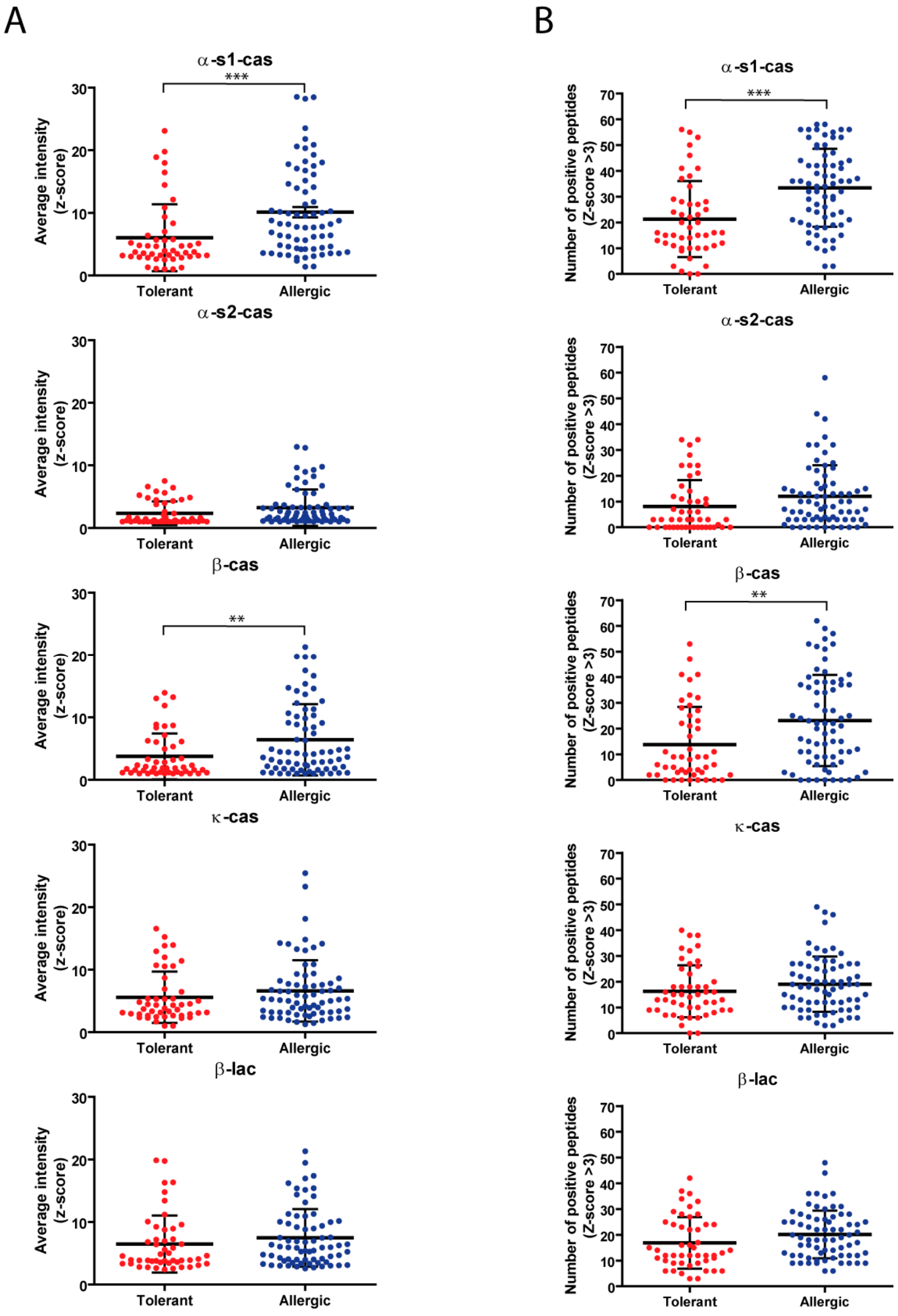

Figure S1. Intensity (A) and diversity (B) of IgE antibodies binding to sequential epitopes of α-s1-, α-s2-, β-, and κ-caseins and β-lactoglobulin, comparing milk tolerant (n= 48) and allergic (n=70) patients at visit 2 (6 months). Each point is an individual patient. Intensity is expressed as the average intensities of peptides recognition (Z-score) from each patient, and diversity is the average number of positive peptides (Z-score<3) recognized by each patient. Mann–Whitney U test p-value (*, $p \leq 0.05$; * *, $p \leq 0.01$; * * *, $p \leq 0.001$).

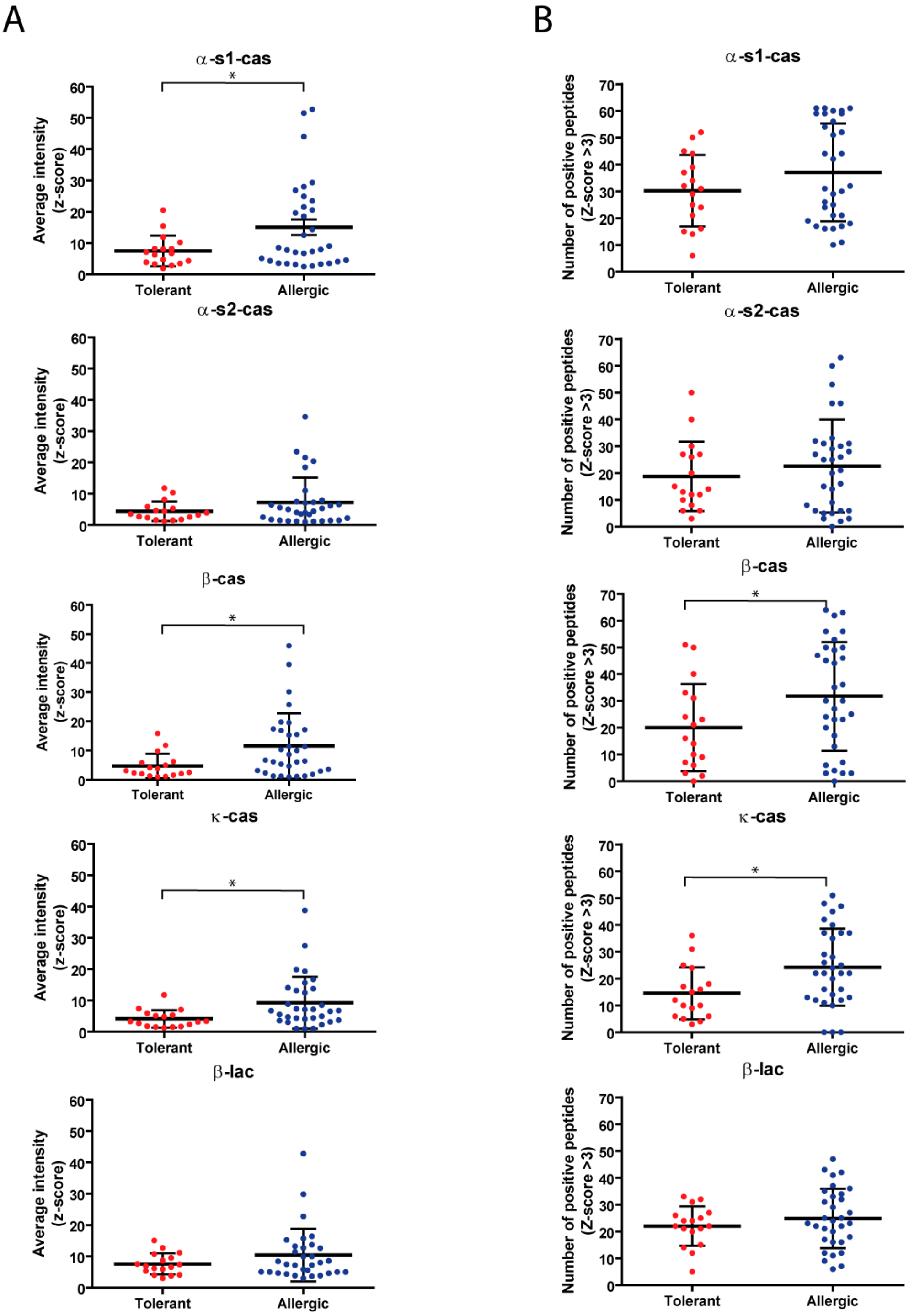

Figure S2. Intensity (A) and diversity (B) of IgE antibodies binding to sequential epitopes of α-s1-, α-s2-, β-, and κ-caseins and β-lactoglobulin, comparing milk tolerant (n= 17) and allergic (n=32) patients at visit 3 (18 months). Each point is an individual patient. Intensity is expressed as the average intensities of peptides recognition (Z-score) from each patient, and diversity is the average number of positive peptides (Z-score<3) recognized by each patient. Mann–Whitney U test p-value (*, $p \leq 0.05$; * *, $p \leq 0.01$; * * *, $p \leq 0.001$).

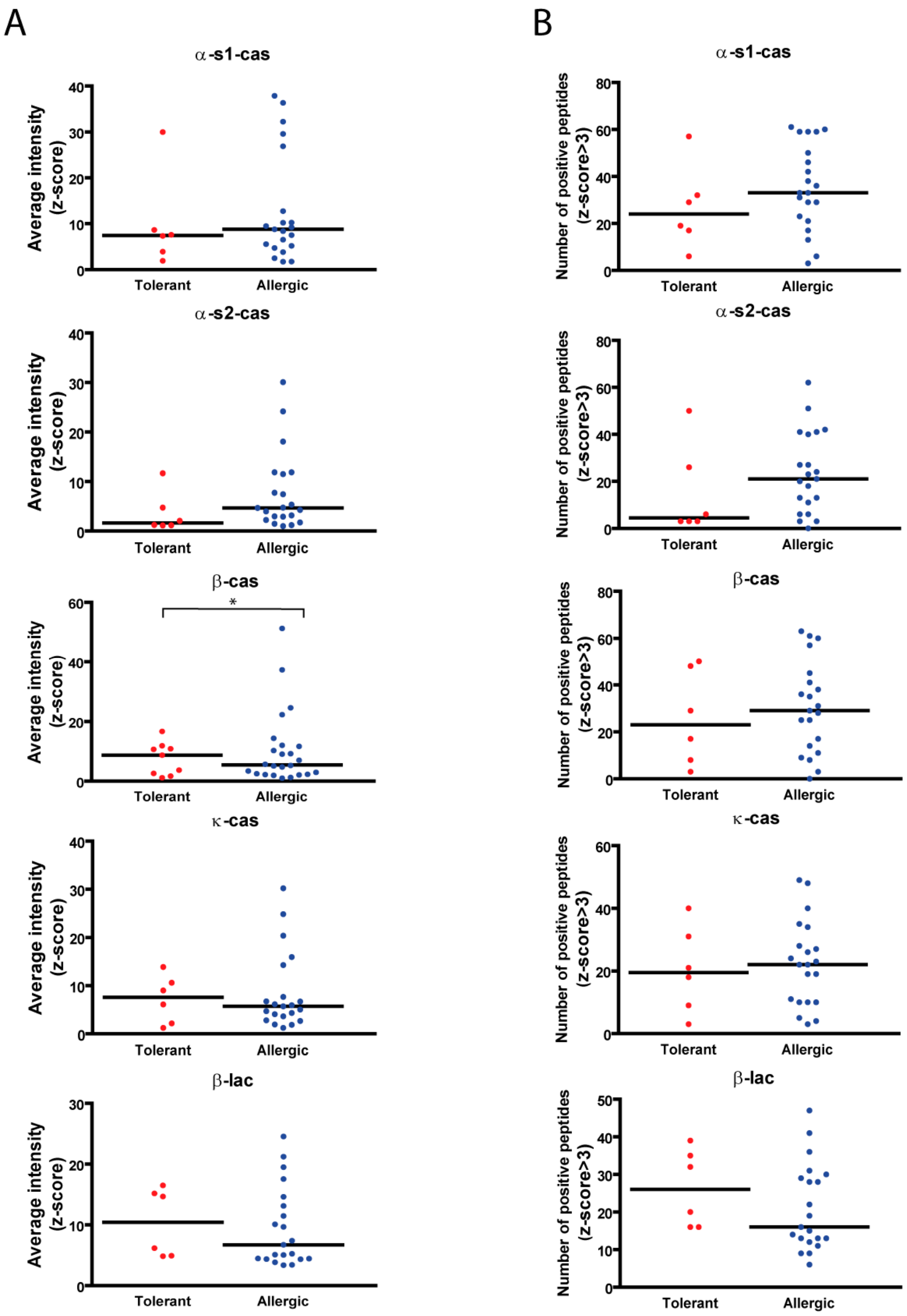

Figure S3. Intensity (A) and diversity (B) of IgE antibodies binding to sequential epitopes of α-s1-, α-s2-, β-, and κ-caseins and β-lactoglobulin, comparing milk tolerant (n= 6) and allergic (n=21) patients at visit 4 (30 months). Each point is an individual patient. Intensity is expressed as the average intensities of peptides recognition (Z-score) from each patient, and diversity is the average number of positive peptides (Z-score<3) recognized by each patient. Mann–Whitney U test p-value (*, $p \leq 0.05$; * *, $p \leq 0.01$; * * *, $p \leq 0.001$).

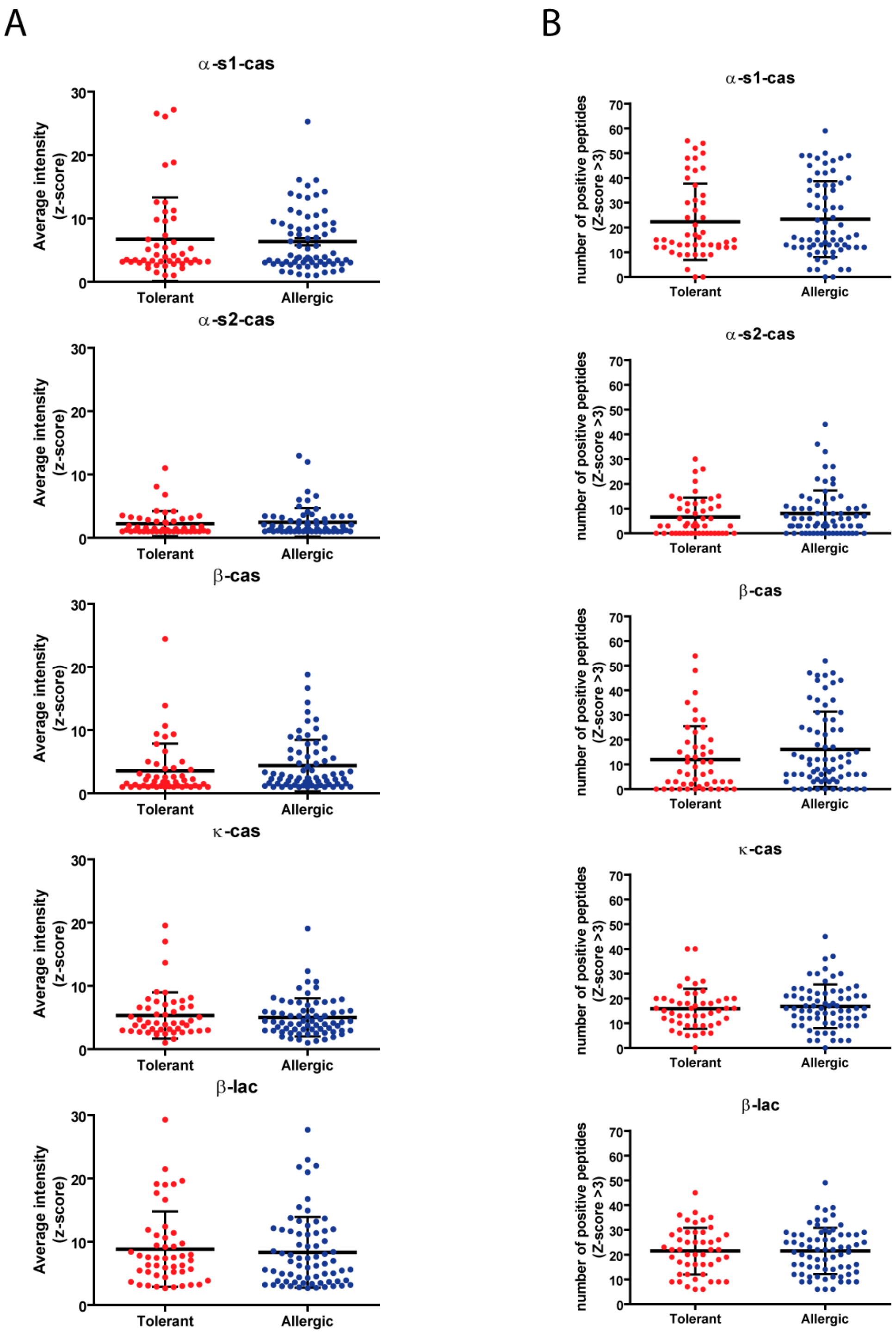

Figure S4. Intensity (A) and diversity (B) of IgE antibodies binding to sequential epitopes of α-s1-, α-s2-, β-, and κ-caseins and β-lactoglobulin, comparing milk tolerant (n= 48) and allergic (n=70) patients at visit 1 (baseline). Each point is an individual patient. Intensity is expressed as the average intensities of peptides recognition (Z-score) from each patient, and diversity is the average number of positive peptides (Z-score<3) recognized by each patient. Mann–Whitney U test p-value (*, $p \leq 0.05$; * *, $p \leq 0.01$; * * *, $p \leq 0.001$).

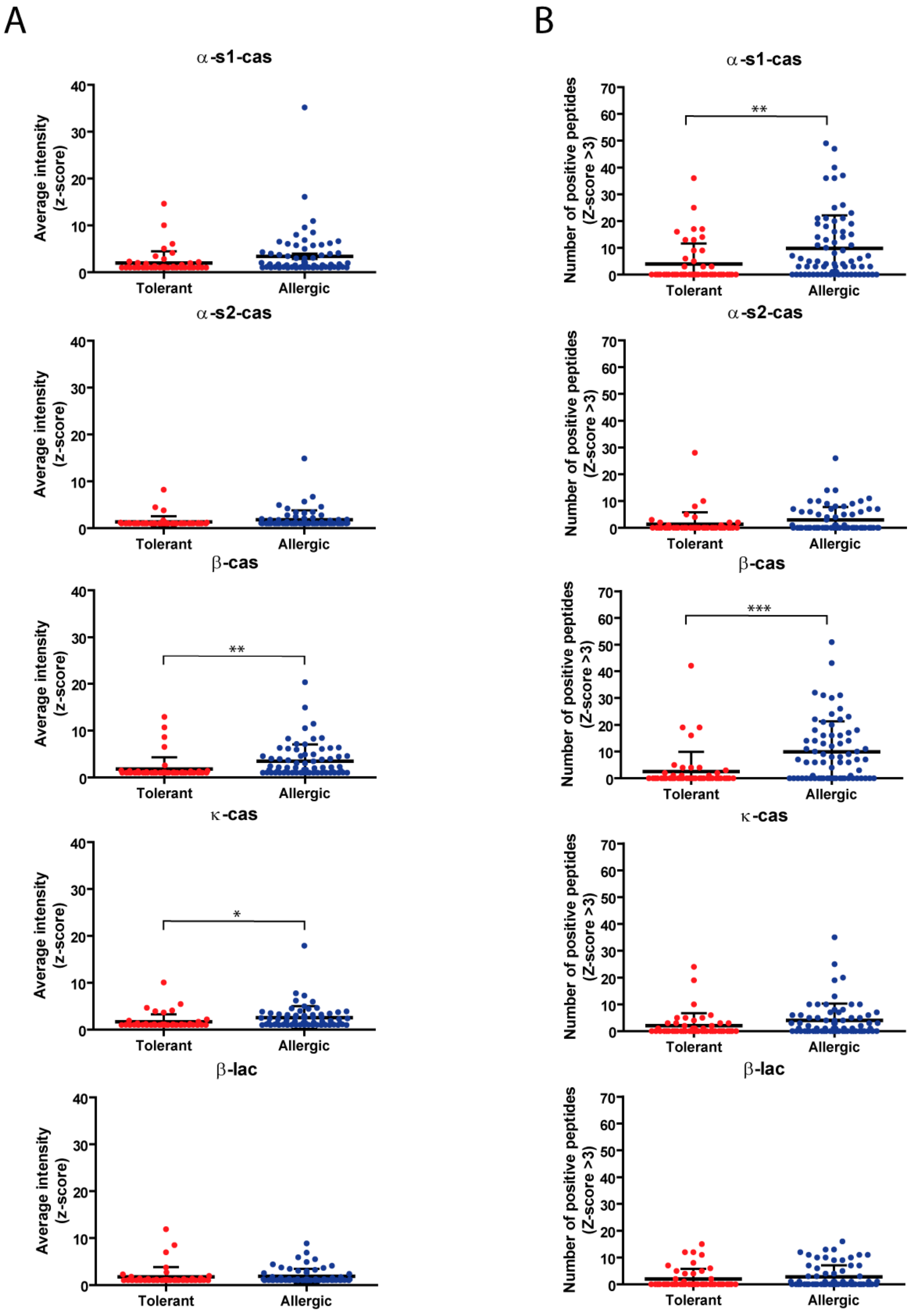

Figure S5. Intensity (A) and diversity (B) of IgG4 antibodies binding to sequential epitopes of α-s1-, α-s2-, β-, and κ-caseins and β-lactoglobulin, comparing milk tolerant (n= 48) and allergic (n=70) patients at visit 2 (6 months). Each point is an individual patient. Intensity is expressed as the average intensities of peptides recognition (Z-score) from each patient, and diversity is the average number of positive peptides (Z-score<3)

recognized by each patient. Mann–Whitney U test p-value (*, p ≤ 0.05; * *, p ≤ 0.01; * * *, p ≤ 0.001).

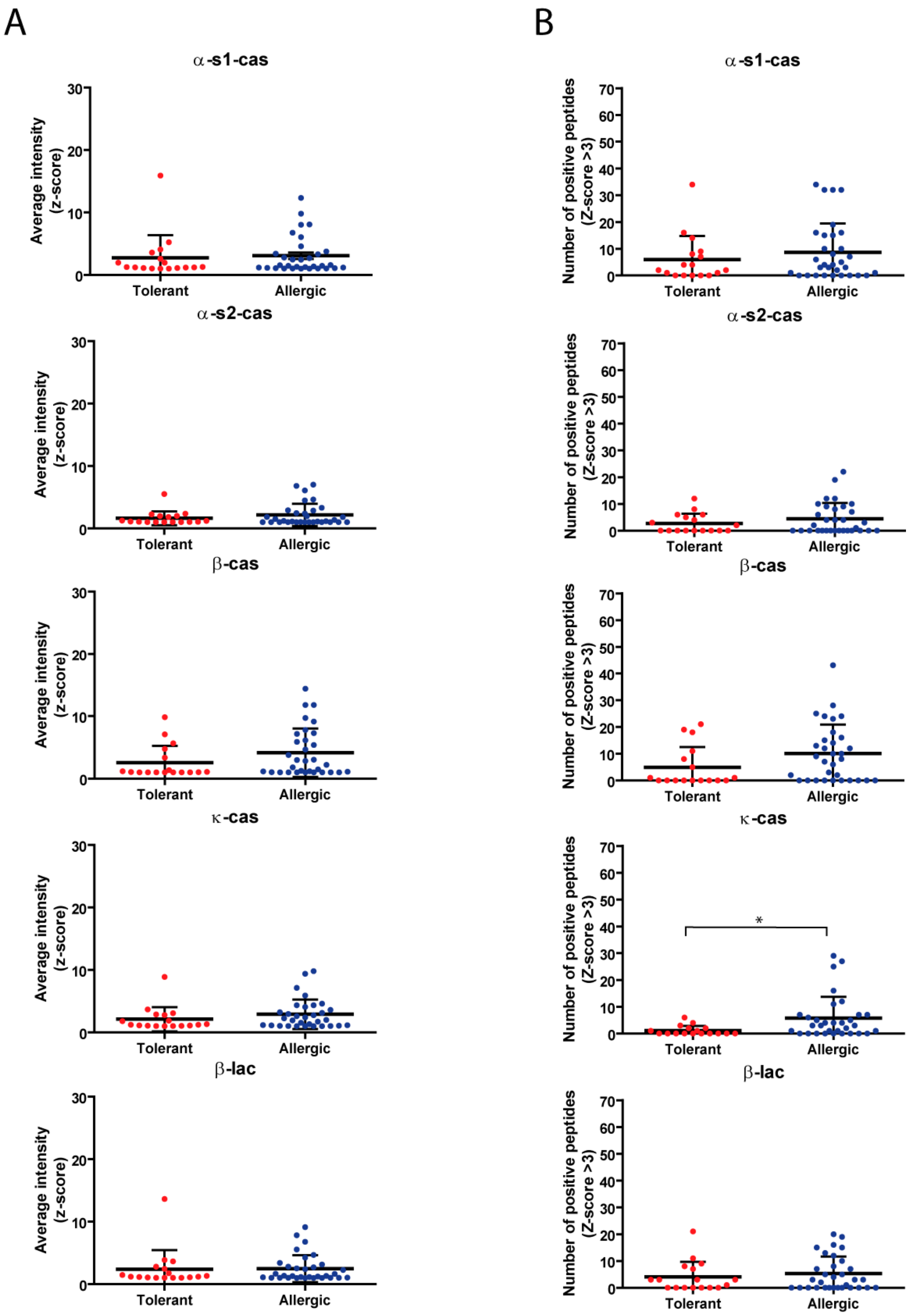

Figure S6. Intensity (A) and diversity (B) of IgG4 antibodies binding to sequential epitopes of α-s1-, α-s2-, β-, and κ-caseins and β-lactoglobulin, comparing milk tolerant (n= 17) and allergic (n=32) patients at visit 3 (18 months). Each point is an individual patient. Intensity is expressed as the average intensities of peptides recognition (Z-score) from each patient, and diversity is the average number of positive peptides (Z-score<3)

recognized by each patient. Mann–Whitney U test p-value (*, p ≤ 0.05; * *, p ≤ 0.01; * * *, p ≤ 0.001).

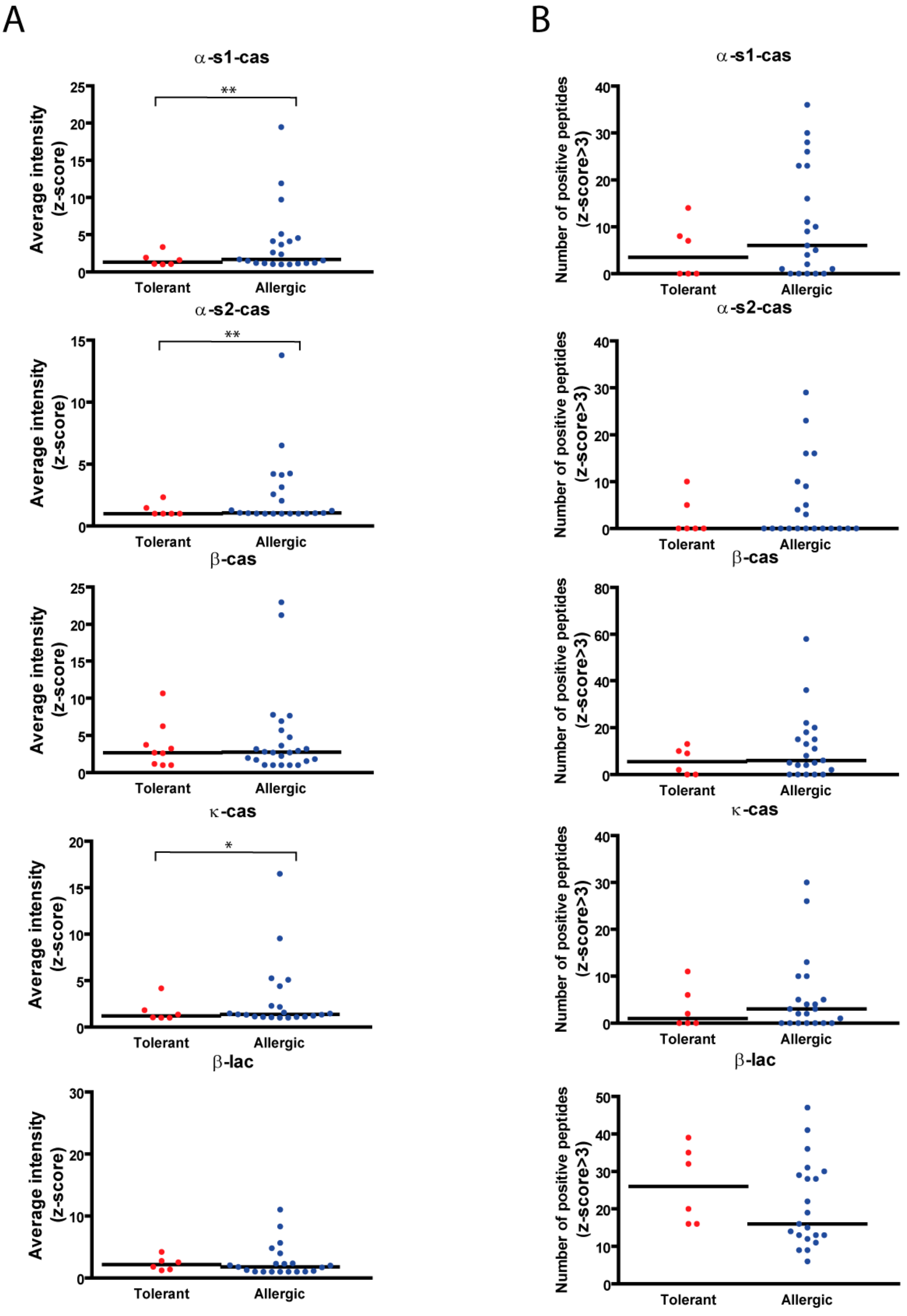

Figure S7. Intensity (A) and diversity (B) of IgG4 antibodies binding to sequential epitopes of α-s1-, α-s2-, β-, and κ-caseins and β-lactoglobulin, comparing milk tolerant (n= 6) and allergic (n=21) patients at visit 4 (30 months). Each point is an individual patient. Intensity is expressed as the average intensities of peptides recognition (Z-score) from each patient, and diversity is the average number of positive peptides (Z-score<3) recognized by each patient. Mann–Whitney U test p-value (*, p ≤ 0.05; * *, p ≤ 0.01; * * *, p ≤ 0.001).

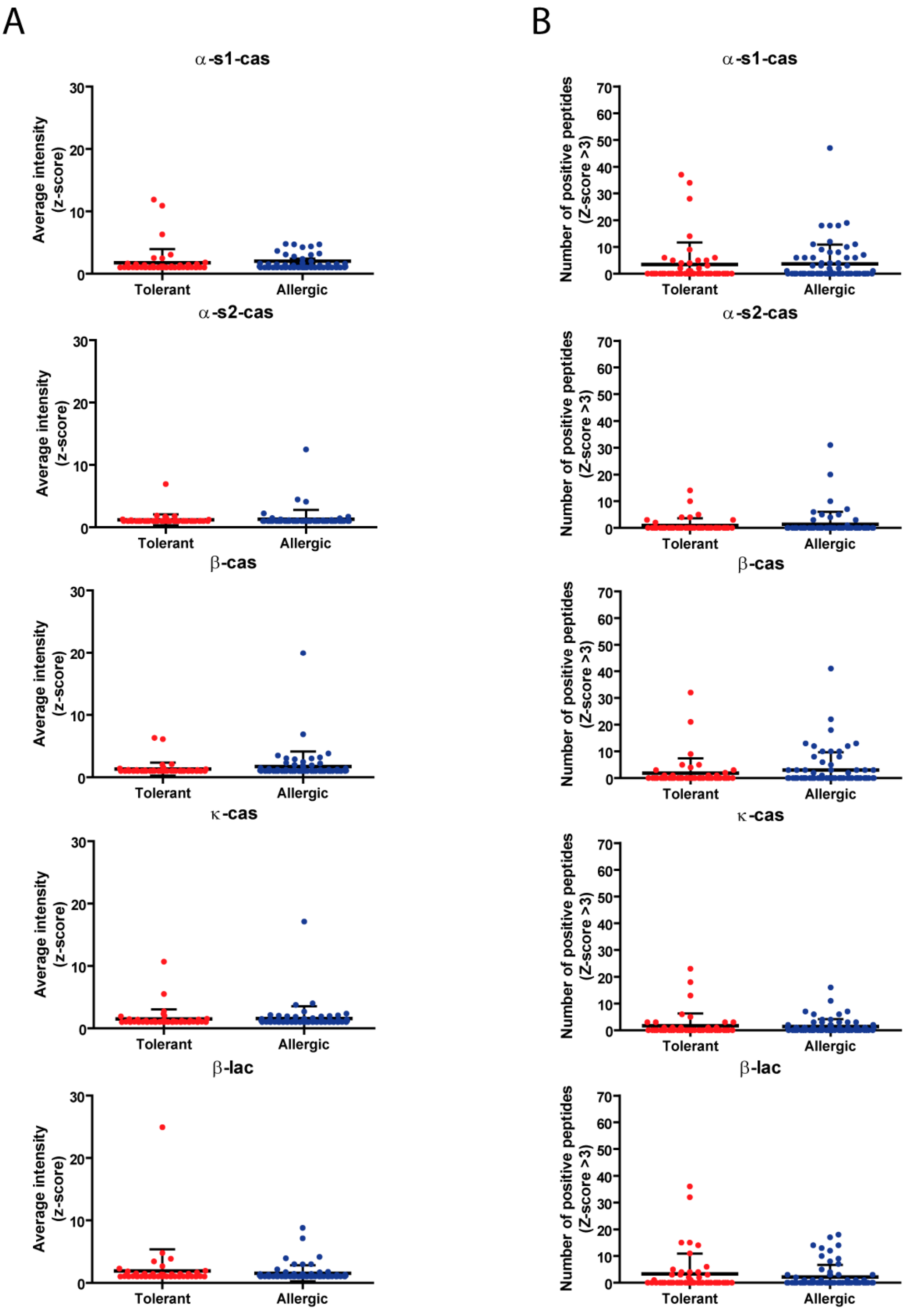

Figure S8. Intensity (A) and diversity (B) of IgG4 antibodies binding to sequential epitopes of α-s1-, α-s2-, β-, and κ-caseins and β-lactoglobulin, comparing milk tolerant (n= 48) and allergic (n=70) patients at visit 1 (baseline). Each point is an individual patient. Intensity is expressed as the average intensities of peptides recognition (Z-score) from each patient, and diversity is the average number of positive peptides (Z-score<3)

recognized by each patient. Mann–Whitney U test p-value (*, $p \leq 0.05$; * *, $p \leq 0.01$; * * *, $p \leq 0.001$).
